# Obsessive-Compulsive Disorder Following Sydenham Chorea: A Case Report

**DOI:** 10.1192/j.eurpsy.2025.704

**Published:** 2025-08-26

**Authors:** M. O. Pires, H. J. Gomes, J. Nunes

**Affiliations:** 1 Unidade Local de Saúde da Guarda, Guarda; 2 Unidade Local de Saúde do Nordeste, Bragança, Portugal

## Abstract

**Introduction:**

Sydenham chorea (SC) is the most common form of autoimmune chorea and is one of the major clinical manifestations of acute rheumatic fever. Although related to group A streptococcal infection, its pathogenesis is not fully understood, and neither is its contribution to the development of Obsessive-Compulsive Disorder (OCD), with hypotheses like autoimmune mechanisms and neuroinflammation being probable. The prevalence of OCD in SC patients is higher than in the general population, with one study reporting obsessive-compulsive symptoms in 24% of SC patients (Moreira *et al*. Parkinsonism Relat Disord 2014;20(2):233-236). Psychiatric symptoms often fluctuate with motor symptoms, but their prognosis is poorly understood.

**Objectives:**

To describe a case of a patient who developed OCD symptoms after SC.

**Methods:**

We report a case of OCD that followed the development of chorea, and we perform a non-systematic review of the literature.

**Results:**

A 34-year-old woman is referred to a Liaison Psychiatry consultation due to cognitive anxiety related to her non-psychiatric history. The patient, with no previous psychiatric history, has a personal history of rheumatic fever (9 years ago) and SC previously evaluated by Neurology consultation where brain magnetic resonance imaging and head computed tomography changes were ruled out, as well as the genetic panel for Huntington disease. No relevant family history. Medicated with sertraline 100mg daily, alprazolam 1mg three times a day, clopidogrel 75mg daily, propranolol 10mg three times a day and atorvastatin 10mg daily. Upon the evaluation, the patient referenced anxiety related to a programmed cardiac surgery and initial and intermediate insomnia. When questioned, the patient reported, after the development of chorea, obsessive ideas and verification rituals/compulsions. These symptoms are time-consuming and cause clinically significant distress and impairment in her work and social functioning. The patient was started on risperidone 0.5mg daily due to its high-potency dopamine D2 receptor blockage that could benefit the chorea and its proprieties in obsessive-compulsive disorder. Six months later, the patient presented an improvement in motor symptoms and a satisfactory Y-BOCS reduction response to the initiated plan.

**Conclusions:**

This case underscores the unusual but significant association between SC and the development of obsessive-compulsive symptoms, contributing to the growing body of evidence that autoimmune processes may underlie neuropsychiatric manifestations such as OCD. The patient’s favorable response to risperidone, a dopamine receptor antagonist, emphasizes the role of dopaminergic dysregulation in both SC and OCD. This case underscores the need for a multidisciplinary approach, including neurology and psychiatry, to manage SC’s neuropsychiatric effects. Further research is needed to clarify how SC relates to OCD and to improve treatment strategies.

**Disclosure of Interest:**

None Declared

